# Synthesis and Application of Silica-Coated Quantum Dots in Biomedicine

**DOI:** 10.3390/ijms221810116

**Published:** 2021-09-18

**Authors:** Xuan-Hung Pham, Seung-Min Park, Kyeong-Min Ham, San Kyeong, Byung Sung Son, Jaehi Kim, Eunil Hahm, Yoon-Hee Kim, Sungje Bock, Wooyeon Kim, Seunho Jung, Sangtaek Oh, Sang Hun Lee, Do Won Hwang, Bong-Hyun Jun

**Affiliations:** 1 Department of Bioscience and Biotechnology, Konkuk University, Seoul 05029, Korea; phamricky@gmail.com (X.-H.P.); hkm3070@icloud.com (K.-M.H.); imsonbs@snu.ac.kr (B.S.S.); susia45@gmail.com (J.K.); greenice@konkuk.ac.kr (E.H.); hilite2201@naver.com (Y.-H.K.); bsj4126@naver.com (S.B.); buzinga5842@konkuk.ac.kr (W.K.); shjung@konkuk.ac.kr (S.J.); 2 Department of Urology, School of Medicine, Stanford University, Stanford, CA 94305, USA; sp293@standford.edu; 3 School of Chemical and Biological Engineering, Seoul National University, Seoul 03080, Korea; san.volcano@gmail.com; 4 Department of Bio and Fermentation Convergence Technology, Kookmin University, Seoul 02707, Korea; ohsa@kookmin.ac.kr; 5 Department of Chemical and Biological Engineering, Hanbat National University, Daejeon 34158, Korea; 6 Department of Nuclear Medicine, College of Medicine, Seoul National University, Seoul 03080, Korea; 7 THERABEST, Co., Ltd., Seocho-daero 40-gil, Seoul 06657, Korea

**Keywords:** quantum dot (QD), surface modification, silica coating, silica encapsulation, bioapplication

## Abstract

Quantum dots (QDs) are semiconductor nanoparticles with outstanding optoelectronic properties. More specifically, QDs are highly bright and exhibit wide absorption spectra, narrow light bands, and excellent photovoltaic stability, which make them useful in bioscience and medicine, particularly for sensing, optical imaging, cell separation, and diagnosis. In general, QDs are stabilized using a hydrophobic ligand during synthesis, and thus their hydrophobic surfaces must undergo hydrophilic modification if the QDs are to be used in bioapplications. Silica-coating is one of the most effective methods for overcoming the disadvantages of QDs, owing to silica’s physicochemical stability, nontoxicity, and excellent bioavailability. This review highlights recent progress in the design, preparation, and application of silica-coated QDs and presents an overview of the major challenges and prospects of their application.

## 1. Introduction

Nanotechnology has made significant contributions to the development of modern society and is currently receiving considerable attention as a result of its potential to break through current stagnation and open up new horizons for technological advancement [1,2,3,4,5,6,7,8,9]. Quantum dots (QDs) are one type of nanomaterial that has been studied intensively over the last 30 years, and both significant and continuous advances in their domain have been made since their introduction in the 1980s [10]. The properties of QDs, which possess particle diameters of 2–10 nm, can be tuned by slightly adjusting their size and composition using the “quantum confinement effect” [11], which dictates that when the radius of a semiconductor particle is smaller than its exciton Bohr radius, the energy spectrum of the particle becomes discrete. This phenomenon results in a unique band gap that is dependent on particle size, and thus QDs can be designed to emit specific fluorescence spectra, ranging from ultraviolet (UV) to near-infrared (NIR) wavelengths, by choosing an appropriate size and base material [12].

Quantum dots offer several advantages over conventional organic fluorescence dyes [13,14,15]. For example, QDs have narrow (typically 25–35 nm full width at half maximum [FWHM]) and symmetric emission spectra, which are more suitable for the simultaneous detection of multiple fluorescence spectra [16,17], and QDs also have broad absorption spectra, which can emit all fluorescent colors of various QDs simultaneously, with only a single excitation source. Moreover, QDs are highly suitable for use as optical probes, owing to their low photobleaching, large molar extinction coefficients, high quantum yields (QYs), and long fluorescence lifetimes when compared to conventional organic fluorescent dyes [10]. Thus, QDs are considered a viable alternative to organic dyes and optical labels and can be used in a wide variety of bioassays [18,19].

However, because high-quality luminescent QDs are typically stabilized using hydrophobic ligands and water is typically used as the basic medium for bioapplications, the hydrophobic surfaces of QDs must be modified [20,21,22]. Three strategies are most commonly used for such hydrophilic modifications [23]. Ligand exchange, for example, involves the replacement of hydrophobic ligands, such as trioctylphosphine (TOP) and trioctylphosphine oxide (TOPO), with hydrophilic ligands that have high affinities to QDs. Meanwhile, amphiphilic combination is based on the hydrophobic attraction between the hydrophobic groups of di- or tri-block copolymers and hydrophobic groups on QD surfaces, and silica (SiO_2_) coating involves covering the surface layer of silanes via crosslinking.

Silica possesses a variety of beneficial characteristics, including facile chemical modification, low cytotoxicity, and excellent chemical stability [24], as well as controllable reactivity, optical transparency, and lack of conductivity. These properties have made silica one of the most widely used elements in nanochemistry, especially for surface modification. The use of silica coating can also resolve some of the key problems related to the practical use of QDs in bioapplications. For example, silica coating can resolve issues related to the photo and colloidal instabilities of QDs in harsh environments and to QD toxicity, since QDs are mainly fabricated using cadmium (Cd), an element that causes itai-itai (ouch-ouch in Japanese) disease [25]. Silica coating can also suppress photoluminescence (PL) bleaching by reducing the photochemical oxidation of cadmium selenide (CdSe) surfaces, which otherwise occurs upon exposure to oxygen molecules [26]. Given these benefits, silica coating is one of the most promising methods for modifying QDs for biological applications.

This paper reviews recent developments in the synthesis and use of silica-coated QDs for biomedical applications. The first part of the paper describes the fabrication and types of silica-coated QDs and the features that differentiate single and multiple silica-coated QDs. The remaining sections describe the bioapplications of nanocomposites based on silica coated QDs. (Note: The core–shell structure which is physically and inherently separated is expressed as a core@shell. The core–shell in QDs is expressed as a core/shell. Core/shell does not mean that the core and shell are separated physically or inherently separated).

## 2. Silica Coating of QDs

### 2.1. Silica-Coated Single QDs

**Figure 1:**
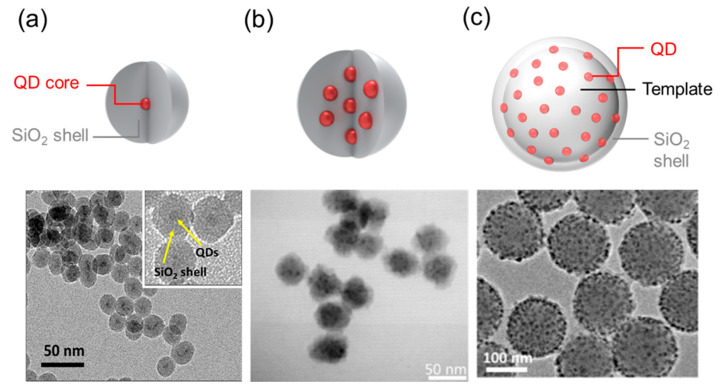
Quantum dot (QD) classification: (**a**) Single QD coated SiO_2_ shell [13], (**b**) Multiply QD-doped SiO_2_ nanoparticle [30], (**c**) Template-based multi-QDs [31].

Individual (i.e., single) QDs can be coated with silica to increase their physical chemical stability (Figure 1a). Considerable improvements have been realized in terms of QD composition, porosity, shape, and metal conjugation to enhance the functionality of QDs in bioapplications.

Single QDs are most commonly silica coated using either the Stöber process or reverse microemulsion [13]. During the Stöber process, which is a sol-gel process based on physical chemistry, monodispersed spherical silica nanoparticles (NPs) are generated by hydrolysis and are subsequently condensed to alkyl silicates (i.e., tetraethyl orthosilicate (TEOS)) in alcoholic solutions, using ammonia as a catalyst, and QDs are used as seeds for silica growth in an ethanol/water mixture [27]. Meanwhile, during reverse microemulsion, which is a water-in-oil process, the template constitutes micelles formed from water, oil, and surfactant. Then, the silica precursor is subjected to hydrolysis and condensation at the water–oil interface or at the water phase, thereby promoting the formation of particles in the template [28].

The Stöber process is generally used to coat relatively large particles (∼50 nm), and the thickness of the silica shells can be tuned by adjusting the silica source and duration of synthesis. In contrast, reverse microemulsion is generally used to coat smaller particles. When several cores are coated as a single particle, the Stöber process is a suitable mechanism whereby the intermixing of all NPs occurs during silica coating, whereas the reverse microemulsion method is probably the principle behind silica coating when several core particles have been inserted into water droplets [29].

### 2.2. Silica-Coated Multi-QDs

#### 2.2.1. Multiple-Doped (“Raisin Bun”-Type) Multi-QDs

The encapsulation of multiple QDs within individual silica particles is an advanced strategy for biomedical applications because of the greater particle brightness. Rogach et al. [32] reported using a sol-gel process to synthesize “raisin bun”-type silica and QD composite (i.e., cadmium telluride, CdTe; CdSe; cadmium sulfide, CdS, or CdSe/Cds) hybrid particles that ranged from 40 to 80 nm in diameter and in which QDs were homogeneously incorporated as multiple cores (Figure 1b). The particle size could be increased to 700 nm using the Stöber process. Even though the PL intensity of the hybrid particles was generally weaker than that of free QDs of equal concentration, each particle emitted intense PL.

Yang et al. [33] also used a sol-gel process and hydrophilic CdTe and hydrophobic CdSe/ZnS QDs to prepare silica QD hybrid particles and reported that individual particles contained multiple QDs (∼7 each). Silica particles with CdTe QDs have PL efficiencies of up to 40% [34].

Even though multiple-doped QDs have considerable advantages for bioapplications, such applications can be inhibited by difficulties in controlling particle size and in maintaining initial PL properties, since QDs are intrinsically unstable in certain chemical environments. Indeed, the PL emissions of QDs inside silica particles can decrease to approximately 20 nm and weaken dramatically to >50 nm. In addition, the silica modification of QD surfaces during incorporation, which requires sophisticated surface chemistry and colloidal stability control, can be inhibited by a variety of technical difficulties [30].

#### 2.2.2. Template-Based Multi-QDs

Template-based multi-QDs (Figure 1c) exhibit substantial potential for bioapplications since the QDs contained in such particles can be positioned in a controlled manner, thereby allowing them to be placed near the particle surface (at depths of <10 nm) and allowing their brightness to exceed that of silica-coated multi-QDs [35]. The process is also advantageous in that it facilitates greater control over particle size.

Woo et al. [36] synthesized colloidal silica microspheres that encapsulated a homogenous QD layer and possessed an outer silica shell. The microspheres were prepared using simple electrostatic interactions between surface-engineered silica and QDs without organic polymers, followed by the Stöber process. The resulting microsphere solution exhibited 3.2 times the PL intensity of QDs with carboxy groups, as well as greater robustness and monodispersity.

Jun et al. [37] developed exceedingly bright QD-embedded silica NPs for biomedical applications. The synthesis of these particles involved preparing highly luminescent (i.e., high PL efficiency) and monodispersed (i.e., narrow PL emission) semiconductor QDs with a thicker ZnS layer of core–shell (CdSe/ZnS) and CdS/ZnS multilayer (CdSe/CdS/ZnS) to protect the QD surface during surface modification. The synthesis process yielded an efficient assembly of ~500 QDs on silica NPs (~150 nm diameter). The resulting multi-QDs exhibited low toxicity and 200-fold stronger PL emission than single QDs and, in particular, did not exhibit any considerable loss in QY. Strong fluorescence signals from SiO_2_@QDs@SiO_2_ NP-tagged cells were monitored and confirmed the suitability of the SiO_2_@QDs@SiO_2_ NPs for in vivo imaging applications.

Nie et al. [38] described QD-doped mesoporous silica beads. The hydrocarbon moiety on the surface of the mesoporous silica was mix with QDs functionalized with a TOP moiety to generate QD-doped mesoporous silica beads. Thereafter, a stable multiple-QD architecture was established via hydrophobic interactions. The loading capacity of the QDs in the mesoporous silica core was affected by diffusion velocity. Finally, 120 million QDs were encapsulated within 5-µm silica beads. This layer-by-layer multi-QD structure exhibited stable optical properties, without bleaching or dissociation [39]. Multiple-doped QDs can overcome some of the issues associated with single QDs (e.g., weak QY) and provide better hydrophilicity via individual surface modification.

In contrast to single QDs, silica-coated multi-QDs can be relatively bulky in size, which can limit their application. However, such particles still exhibit adequate brightness and high PL efficiencies while maintaining the advantages of silica-coated single QDs (e.g., hydrophilic properties and convenient surface treatment). The steps involved in QD-doped multi-QDs are relatively straightforward. However, it remains challenging to adjust QD size and to control the number of QDs in each particle. On the other hand, template-based multi-QDs are highly adaptable and brighter than QD-doped multi QDs, and it is relatively easy to control the number of QDs in each particle.

### 2.3. Silica-QD Hybrids with Functional Materials

In addition to the morphological transformations of QDs, a variety of functional materials, such as plasmonic NPs, magnetic particles, and conductive polymers, have been combined with QDs. The functional materials are used to improve QD performance or to provide additional functionalities while maintaining particle performance. Numerous experimental studies have introduced plasmonic NPs to QDs to improve the QD characteristics, such as their optical properties, chemical and physical resistances, and bioimaging resolution, all of which are essential prerequisites for bioapplications.

Ji et al. [40] reported the encapsulation of a single QD at the center of a small bead of amorphous silica and the chemical deposition of an Au nanoshell onto the silica surface. In such a hybrid system, the Au nanoshell acts both as a barrier, which considerably improves photochemical robustness, and as a plasmon resonator, which increases both the excitation field seen by the QDs and the local density of states. Thus, the Au nanoshell functions as a shield, protecting QD fluorescence and enhancing QD resistance against high-power photoexcitation and high-energy electron beams.

Serrano et al. [41] developed a multistep process for synthesizing hybrid superstructures that included QD cores with dense layers of Au NPs separated by a silica shell. This architecture facilitated the regulation of QD–metal interactions by controlling the thickness of the dielectric spacer, and shell thickness was optimized to the nanometer scale to enhance PL. Further characterization of the emission in a single-particle regime revealed that smaller acquisition times are needed to obtain high-quality images of brighter particles. Notably, in contrast to the as-prepared Au NPs, which were too small to sustain localized surface plasmon resonance, QD@SiO_2_@Au NPs exhibited broadened bands, with a maximum at 556 nm, attributed to the plasmon coupling of Au particles and the subsequent generation of electromagnetic intercoupling (i.e., hot spots).

**Figure 2:**
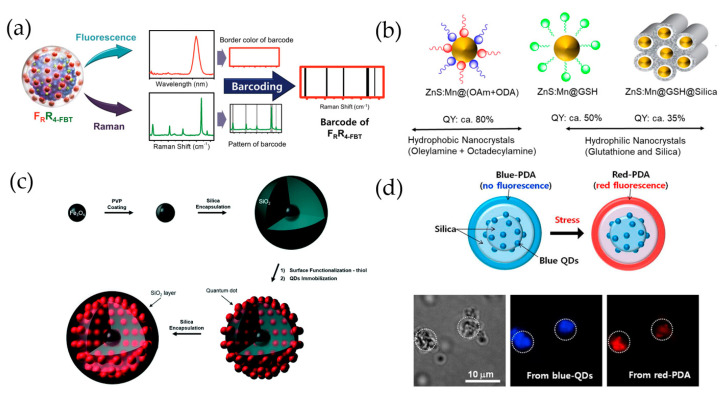
(**a**) Fluorescence-SERS QD-embedded silver bumpy nanoprobes, which have a SiO_2_@AgNS@SiO_2_@QD_2_@SiO_2_ structure. Forty-five different dual-modal nanoprobes were prepared from silica-coated silver bumpy nanoshells (AgNS@SiO_2_) with 15 different types of Raman label compounds and 3 types of QDs (red, green, and blue) [42]. (**b**) Mn-doped ZnS (ZnS:Mn) QDs combining dual ligands (oleylamine and octadecylamine), hydrophilic glutathione (GSH) ligands, and silica for biomedical application. Adapted with permission from ACS Appl. Nano Mater. 2020, 3, 3, 3088–3096. Copyright 2020 American Chemical Society [49]. (**c**) Dual-functional nanoprobe composed of an iron oxide core surrounded by QD-embedded silica NPs [44]. (**d**) SiO_2_@QDs@PDA NPs for label-free, multiplexed detection of biological molecules [47].

Many studies have aimed to improve QD functionality. For example, Cha et al. [42] reported the development of fluorescence- surface-enhanced Raman spectroscopy (SERS) QD-embedded silver bumpy nanoprobes with a SiO_2_@AgNS@SiO_2_@QD_2_@SiO_2_ structure (Figure 2a). These authors prepared 45 dual-modal nanoprobes from silica-coated silver bumpy nanoshells with 15 different types of Raman label compounds and three types of QDs (red, green, and blue) for high-throughput multiplex analysis. In this study, a silica shell (~20 nm) was used between the AgNS and QD layers to separate the introduced QD fluorophores, thereby preventing quenching due to their mutual proximity.

Nontoxic Mn-doped ZnS QDs (ZnS/Mn) can be used as nanoprobes for biomedical applications (Figure 2b). Ang et al. [43] demonstrated that alkylamine ligands with carbon chain lengths of C18, such as oleylamine (OAm) and octadecylamine (ODA), promote the diffusion of Mn from the surface of ZnS nanocrystals to their interior. In this study, the ZnS/Mn@(OAm+ODA) nanocrystals fabricated by combining dual ligands (e.g., OAm and ODA) could reach up to 80% QY. When organic-soluble doped ZnS/Mn nanocrystals were replaced with hydrophilic glutathione (GSH) ligands, the QY of ZnS/Mn@GSH was maintained at approximately 40–50%, but when an additional silica layer was added (ZnS/Mn@GSH@SiO_2_), the QY was reduced to 35–40%. The ZnS/Mn nanocrystals were made water-soluble by addition of the GSH ligand, and the silica-coated particles demonstrated potential for cancer theranostic applications through in vitro cell-labeling and drug-release studies.

Lee et al. [44] reported that QD-embedded silica NPs with iron oxide NP cores were a dual-functioning material (Figure 2c), owing to their superparamagnetic and highly fluorescent properties. To generate these particles, multiple QDs were immobilized onto the silica surface of the Fe_3_O_4_@SiO_2_ particles that were intended to be sorted by either fluorescence flow cytometry or a magnetic field.

Lin et al. [45] also fabricated multifunctional magnetic resonance/optical NPs from silica-coated CuInS_2_/ZnS NPs through the covalent attachment of a Gd^3+^ complex via carbodiimide chemistry. The dual-modality NPs exhibited negligible cytotoxicity, with >80% cell viability for the human pancreatic cancer cell line (BxPC-3 cells) after 24 h, and in regard to both optical and magnetic resonance imaging (MRI), were successfully applied to cell culture in aqueous solutions.

Jinadasa et al. [46] fabricated phenobarbital-containing polymer/silica-coated Mn/ZnS QDs (Ph-QDs) for the selective detection and quantification of Hg species in fishery products using a room-temperature phosphorescence quenching assay. To synthesize Ph-QDs, Mn/ZnS QDs were encapsulated in silica using TEOS and modified with phenobarbital-containing polymers that have demonstrated an affinity for inorganic and organic Hg species using (3-aminopropyl triethoxysilane). Even though QD probes are usually only selective for a single Hg species, usually Hg(II), the Ph-QDs prepared in this study provided high selectivity for both Hg(II) and MeHg, and the proposed phosphorescence-based Ph-QDs method gave results similar to those obtained using more sophisticated analytical techniques (e.g., ICP-MS), and provided a sufficiently low limit of detection for total Hg in fish samples.

In another study [47], QD-assembled silica NPs were designed for label-free, multiplexed detection of biological molecules and were produced by adding a polydiacetylene (PDA) supramolecule to the NP surface (Figure 2d). Two types of QD-assembled silica NPs (SiO_2_@QDs NPs) were prepared and coated with PDA supramolecules via the photo-induced polymerization of 10,12-pentacosadiynoic acid. The as-prepared SiO_2_@QDs@PDA NPs exhibited discrete QD PL for the encoding and fluorescence of PDA for sensing a target without interference or overlap.

Mesoporous silica shells are often used to fabricate multimodal NPs, which are synthesized by coating core particles that include NPs with different functions. Because a relatively large number of NPs can be placed in each pore, it is possible to generate multimodal NPs that are superior to conventional NPs.

Lixin et al. [48] designed a uniformly shaped multifunctional nanoprobe platform that consisted of mesoporous silica with an intermediate layer that contained CdTeS QDs and a silica shell with a superparamagnetic ferroferric oxide (Fe_3_O_4_) cluster as the core. Since CdTeS QDs function as a fluorescence labeling agent and Fe_3_O_4_ cluster cores exhibit magnetism, the nanoprobe particles exhibited superparamagnetism at room temperature, thereby enabling magnetic separation and fluorescence imaging. In this study, for example, the pores were modified using folic acid so that the probe was absorbed by the cancer cells. The photothermal effect of the Fe_3_O_4_ cluster core can also be used in the treatment of cancer cells. Thus, multifunctional nanoprobes can be used for fluorescence detection, magnetic separation, and photothermal therapy.

Peng et al. [49] synthesized functional NPs capable of both fluorescence and MRI bioimaging by placing Mn-doped ZnSe QDs in mesoporous silica NPs (MSNs) with large pores (MSN@QDs) that facilitated dense QD placement. The NP enrichment factor of these particles was ~143, which indicated very strong fluorescence. MSN has amine-modified pores, is biocompatible, and has high photostability. Moreover, compared to existing s-QD NPs, MSN@QD NPs exhibit greater brightness of fluorescence and strong magnetic signals for both in vitro and in vivo MRI and imaging, thereby showing promise for the diagnosis of cancer.

Damien et al. [50] coated magnetic iron oxide cores (18 nm diameter) with a large-pored stellate mesoporous silica (STMS) shell to produce uniform-sized IO@STMS NPs. The pores were then filled with CdSe/ZnS QD NPs to enhance fluorescence, and the particles were coated with human serum albumin to improve biocompatibility, and thus facilitate photothermal therapy, MRI, and fluorescence imaging. The functionalities of this particular nanoprobe as a fluorescent imaging and MRI contrast agent were confirmed using nuclear magnetic resonance (NMR) relaxometry and spectrofluorometry, and target cells were killed (45%) when chemo- and photo-thermal therapies were combined under clinical conditions (117 G, 100 kHz).

## 3. Bioapplications of Silica-Coated QDs

### 3.1. QDs as Flourescence Labels

Silica-coated QDs have been actively utilized for selective labeling in a variety of in vitro bioassays, including the lateral flow assay (LFA), quantum dot-linked immunosorbent assay (QLISA), magnetic bead-QD assay, multiplex flow cytometric immunoassay, electrochemical immunoassay, and paper-based in vitro assay.

Xu et al. [51], for example, fabricated biocompatible and robust InP/GaP/ZnS QD@SiO_2_ particles for a sensitive C-reactive protein (CRP) immunoassay. In this study, oleic acid (OA) and 1-octanethiol (OT)-capped hydrophobic InP/GaP/ZnS QDs were pre-silanized with catalytic-free TEOS hydrolysis, and outer SiO_2_ shells were generated on the QD surfaces in a reverse microemulsion. The QY of the original InP/GaP/ZnS QDs was 72%, and the resulting InP/GaP/ZnS QD@SiO_2_ NPs possessed a high QY (65%) despite the introduction of a silica shell. After synthesis, the InP/GaP/ZnS QD@SiO_2_ NPs were coupled with a CRP antibody probe and used for QLISA. Low-toxicity InP-based QDs are less advanced than Cd-based QDs because of their stability and QY in aqueous solutions. The QLISA based on InP/GaP/ZnS QD@SiO_2_ exhibited high sensitivity to CRP and was similar in performance to silica-encapsulated Cd-based QDs.

Jo et al. [31] reported the sensitive detection of the H1N1 virus using a sandwich immunoassay of capture antibody-magnetic beads (M-beads) and detection antibody-QD_2_. In this study, the PL of a silica-coated cluster of 400–500 CdSe@ZnS QDs on a silica core (QD_2_) was several hundred times greater than that of individual QDs. The QD_2_s were also conjugated with anti-virus antibodies and used as a fluorescent probe for immunoassays. The polystyrene-based M-beads, with uniform size and superparamagnetic properties, were reacted with H1N1 virus and QD_2_ probe and then used to separate the target virus from other non-target analogs. The detection system exhibited a very low limit of detection for H1N1 and was 2100 times more sensitive than the conventional hemagglutination method.

Similar QD structures have been applied to multiple QD-embedded nanobeads (SiO_2_@QD@SiO_2_-COOH) [52], which have 50 times greater PL than single QDs but a similar QY. Such nanobeads exhibit high chemical and physical stabilities that can be demonstrated under acidic and high-temperature conditions, respectively, and thus could be used to develop highly sensitive and quantitative immunoassays. Indeed, a QLISA-based CRP immunoassay that was developed using SiO_2_@QD@SiO_2_-COOH nanobeads exhibited a broad dynamic range (0.5–1000 ng/mL) and high sensitivity (detection limit: 0.32 ng/mL).

**Figure 3:**
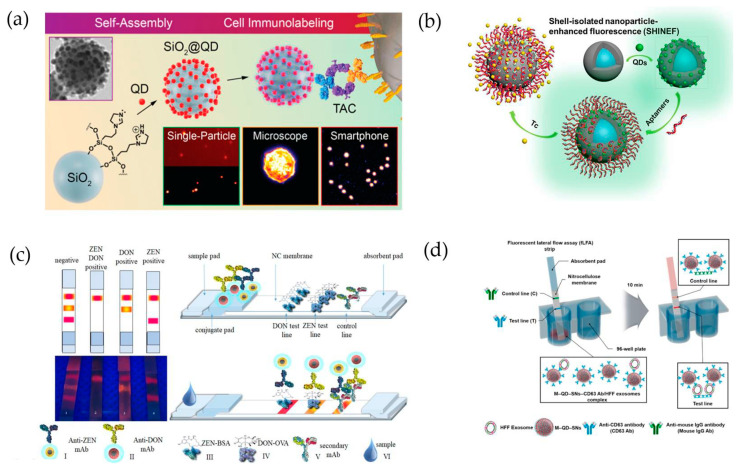
(**a**) QD-introduced self-assembly procedure into silica NPs (SiO_2_@QD-TACs) and cell immunolabeling [53]. (**b**) QD-embedded silica-coated Ag NPs (Ag@SiO_2_/QDs) and attached aptamer (Ag@SiO_2_/QDs-Apt). Tc detection using shell-isolated NP-enhanced fluorescence (SHINEF) [54]. (**c**) Detection of zealralenone (ZEN) and deoxynivalenol (DON) using LFIA with QD@SiO_2_ [55]. (**d**) Detection of human foreskin fibroblast exosomes using fLFA with M-QD-SNs [56].

To develop a more efficient biomarker detection system based on QDs, Ghinwa et al. [53] synthesized QD-assembled SiO_2_ nanostructures (SiO_2_@QD), which were loaded with a larger supra-NP assembly (Figure 3a). Multiple QDs were introduced by spontaneous affinity interactions between imidazoline-functionalized SiO_2_ NPs, followed by conjugation of tetrameric antibody complexes (TACs). The prepared SiO_2_@QD@TACs were able to detect the surface biomarker of breast cancer (SK-BR3) cells. Because of the high PL, SiO_2_@QDs provided a higher sensitivity and signal-to-noise ratio, and could thus be useful for cancer diagnostics with microscopy or smartphone-based fluorescence imaging.

Interestingly, QD-based nanostructures can also be used as detection probes for Tc, which is a broad-spectrum tetracycline antibiotic. Xueqing et al. [54], for example, used aptamer-labeled fluorescent nanoprobe-integrated shell-isolated NP-enhanced fluorescence spectroscopy (SHINEFs) for Tc detection (Figure 3b). The authors introduced silica-coated Ag (Ag@SiO_2_) NPs to produce fluorescence (Ag@SiO_2_/QDs), and an aptamer was added to the QD surfaces (Ag@SiO_2_/QDs-Apt) to ensure highly sensitive detection. The maximum SHINEFs was a 9-nm silica spacer. The linear dynamic range and detection limit of the probe were 0.2–400 μM and 16.2 nM, respectively, and the recovery rate was 96.8–91.8% when used to detect Tc in milk. These results demonstrate that QD-fabricated nanoprobes could be used as other common substances.

Goryacheva et al. [55] synthesized silica-coated CdSe/CdS and CdSe/Cds/Zns QDs to use as fluorescent nanoprobes in a multicolor lateral flow immunoassay (LFIA) that was designed to simultaneously detect multiple mycotoxins (Figure 3c). The CdSe-based QDs were synthesized using a hot injection method and coated with a hydrophilic silica shell using silanization through reverse microemulsion. The synthesized CdSe-based QDs-SiO_2_ exhibited a PL QY of up to 70% in aqueous media. As optimized LFIA probes, red-emitting CdSe5.0 nm/CdS6ML-SiO_2_ (PL QY: 63%) and orange-emitting CdSe3.6 nm/CdS6ML-SiO_2_ (PL QY: 51%) were conjugated to separate antibodies. The resulting assay was capable of rapidly screening for zealralenone and deoxyniva-lenol, which are mycotoxins produced by *Fusarium* fungi, in naturally contaminated corn and wheat samples.

Exosomes, which are a type of extracellular vesicle, are receiving great attention as biomarkers for disease diagnosis (Figure 3d). Indeed, colorimetric-based LFIAs have been developed to detect exosomes. However, such assays are limited by their relatively low quantification capabilities. Accordingly, Kim et al. [56] proposed that multi-QDs embedded in silica-encapsulated NPs (M-QD-SNs; with a SiO_2_@QDs@SiO_2_ structure) could be used to detect exosomes in human foreskin fibroblasts (HFFs). As a fluorescent probe, the M-QD-SNs, which were coated with a thin silica shell to facilitate surface modification, were conjugated to an anti-CD63 antibody and applied to fLFIA. The M-QD-SNs exhibited a high PL intensity, and the QY was relatively unaffected by either surface modification or antibody conjugation, retaining PL emissions that were hundreds of times greater than those of single QDs. Using this fLFA, the abundance of exosomes could be rapidly and quantitatively measured.

Wu et al. [57] studied QD-based LFIA for the detection of cardiac troponin I (cTnI), which is a biomarker for myocardial infarction and acute myocardial infarction. The CdSe/ZnS QD-based nanoprobes were developed by encapsulating QDs in silica (QD@SiO_2_). As a result, the fabricated QD@SiO_2_ NPs exhibited water-soluble properties, and the resulting LFIA system was capable of rapidly (in 10 min) recognizing cTnI with high sensitivity (detection limit: 5.6 × 10^−3^ ng/mL), broad linear range (0.8–200 ng/mL), and high precision (coefficient of variation: <10%). This LFIA strip has also been successfully applied to human serum.

QD-based LFIAs have also been used for bacterial detection. Bo et al. [58] introduced QDs into a SiO_2_ core using an interlayer of cationic polyethyleneimine (PEI) and carboxylated the QDs onto the PEI to form a nanocomposite shell, thereby promoting dispersibility. The resulting LFIA strip that incorporated the PEI-interlayered SiO_2_-core QD-shell nanocomposite (SiO_2_@PEI-QDs) probe yielded strong fluorescent signals for the detection of *Salmonella typhimurium* and was capable of detecting bacteria concentrations as low as 5 × 10^2^ cells/mL.

Beloglazova et al. [59] described the synthesis of silica-coated liposomes that were loaded with QDs and suggested the probes as prospective immunoassay labels. The silica-coated package material for the encapsulation of water-insoluble QDs allowed the particles to bioconjugate and enhanced the stability of liposomes against fusion and internal leakage during storage, transportation, and application. Silicanized liposomes were then used for the sensitive multiplex immunochemical determination of two analytes (mycotoxins zearalenone and aflatoxin B1), and silanized liposomes loaded with different-colored QDs were able to simplify the evaluation of multiple assays.

Goftman et al. [60] fabricated bright and stable fluorescent biolabels for the immunoassay detection of the mycotoxin deoxynivalenol in food and feed. In the study, CdSe/CdS/ZnS core-shell QDs were encapsulated in silica NPs using a water-in-oil reverse microemulsion process. To assess the bioapplicability of the labels, optical properties and stabilities were characterized for silica-coated QDs that were modified with amino, carboxyl, and epoxy groups and stabilized using polyethylene glycol (PEG) fragments. Co-condensation techniques were developed to retain 80% of the initial fluorescent properties and yielded stable fluorescent labels that could be easily activated and bioconjugated. Furthermore, the modified QD@SiO_2_ particles were conjugated efficiently with antibodies and used successfully as novel labels in microtiter plate-based immunoassays and quantitative column-based rapid immunoassays for the detection of deoxynivalenol.

### 3.2. FRET-Based Assay for Biomolecule Sensing

Förster resonance energy transfer (FRET), which is a non-radiative energy transfer between donor fluorophores and acceptor quenchers, has been used as an analytical approach in a variety of fields and especially in methods of biomolecule sensing, such as immunoassays and molecularly imprinted polymer (MIP)-based sensing. Interestingly, QDs can be used as donor fluorophores, and during the synthesis of the nanocomposites for use as FRET sensors, the silica layered structure of QDs can provide a template for surface modification with specific receptors. Indeed, several silica-coated QD-based FRET sensors have been reported [61].

**Figure 4:**
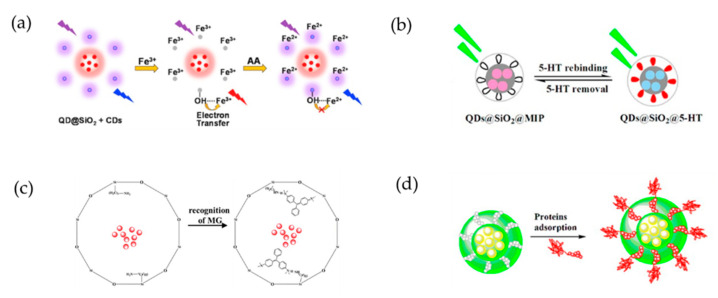
(**a**) Ratiometric fluorescence detection of ascorbic acid (AA) based on the “OFF” and “ON” step of a nanoprobe that contains a blue-emitting carbon dot (CD) and red-emitting, silica-coated QDs (QDs@SiO_2_) [62]. (**b**) Fluorescence detection of serotonin (5-HT) using hybrid QDs, silica, and molecularly imprinted polymers (QDs@SiO_2_@MIPs) [63]. (**c**) Selective recognition of malachite green (MG) using MIP-coated QDs [64]. (**d**) Specific recognition and fluorescence quantification of bovine serum albumin (BSA) using epitope MIP (EMIP) coated QDs [65].

Widely reported fluorescence detection methods that are used for monitoring ascorbic acid (AA) for food quality and healthcare are generally based on “on” or “off” systems (Figure 4a). Zhao et al. [62] developed a ratiometric fluorescent test paper that was based on fluorescent nanoprobes that consisted of blue-emitting carbon dots (CDs) and red-emitting silica-coated QDs (QDs@SiO_2_). In this system, the fluorescence of the blue-emitting CDs was quenched in the presence of Fe^3+^ and was restored as the concentration of AA increased and Fe^3+^ was reduced to Fe^2+^. Meanwhile, the fluorescence of the red-emitting QD@SiO_2_ NPs was used as a stable internal standard, and the CD-QD@SiO_2_-Fe^3+^ probe detected AA with high selectivity because it was not affected by the coexistence of other compounds or metal ions. Thus, a distinct color change, from red to blue, could be observed with the naked eye as the concentration of AA increased.

Wang et al. [63] proposed a fluorescence method for detecting serotonin (5-HT) that was based on a layer-structured probe composed of Mn-doped ZnS QDs, silica nanoparticles, and molecularly imprinted polymers (QDs@SiO_2_@MIPs; Figure 4b). A complex of QDs@SiO_2_@MIPs and 5-HT was formed by hydrogen bonding between the amino and hydroxyl groups. When 5-HT rebinding occurred, the transfer of energy from the QDs to the complex induced quenching of the fluorescence. The composite exhibited good selectivity, with an imprinting factor of 5.96, correlation coefficient of 0.9928 at 50–500 ng/mL, and a limit of detection of 0.69 ng/mL. The probe required a simple preparation process and demonstrated high sensitivity, good selectivity, low detection limit, and short analysis time. The proposed composite appears to be highly suitable for detecting 5-HT in human serum.

Wu et al. [64] also reported the use of an MIP-coated QD structure as a fluorescent probe for the selective detection of malachite green (MG; Figure 4c). In this FRET system, MG quenched the fluorescence intensities of the MIP-coated QDs, and the system exhibited a selective signal response toward MG and a limit of detection of 12 μg/kg. Furthermore, the system was successfully applied to the detection of MG in spiked fish samples. The MIP-coated QD recoveries ranged from 94.3% to 109.5%, with relative standard deviations <4.8%.

Yang et al. [65] reported the development of a nanocomposite with an epitope molecularly imprinted polymer (EMIP) for the specific recognition and direct fluorescent quantification of bovine serum albumin (BSA; Figure 4d). The EMIP film was formed by the polymerization of 3-aminopropyl triethoxysilane, as a functional monomer, on the surface of silica nanosphere-embedded CdTe QDs, and a synthetic peptide derived from the surface-exposed C-terminus of BSA (residues 599–607) was used as the EMIP template molecule. The resulting EMIP film selectively captured BSA via specific recognition cavities. The EMIP-coated QDs were used as template nanospheres for the direct fluorescence quantification of BSA. The EMIP-coated QDs exhibited much greater imprinting and adsorption than BSA MIP (BMIP)-coated QDs. The analytical performance of the EMIP-coated QDs was assessed by evaluating the ability of the QDs to detect BSA in a bovine calf serum sample, and satisfactory results were obtained during the separation of BSA from the bovine blood sample.

To development a system for the selective detection of thrombin, Park et al. [66] coated Fe_3_O_4_ nanocrystals with a QD-layered silica composite and then introduced a thrombin-binding aptamer that was capable of hybridizing quencher DNA (qDNA) to the outer silica shell. During qDNA-aptamer hybridization, fluorescence quenching occurred as a result of energy transfer between adjacent QDs and qDNA. However, because the binding of thrombin displaced qDNA, the fluorescence signal was restored in proportion to thrombin concentration. In addition, the composite’s Fe_3_O_4_ nanocrystal core resulted in superparamagnetic properties that facilitated magnetic capture. During the analysis of thrombin by simple injection, the proposed method exhibited improved sensitivity, with a detection limit of 0.35 nM.

### 3.3. QDs for Imaging

#### 3.3.1. In Vitro Cell Imaging

Quantum dots can also be used for the imaging of cells and tissues. However, a QD imaging system might require specific functionalities different from those used for molecular detection. The excellent optical properties of QDs (e.g., extreme brightness, high photostability, continuous absorption, and narrow emission bandwidth) make them ideal optical labels for the development of QD-based immunofluorescence (IF) imaging, especially for multiplex biomarker detection.

**Figure 5:**
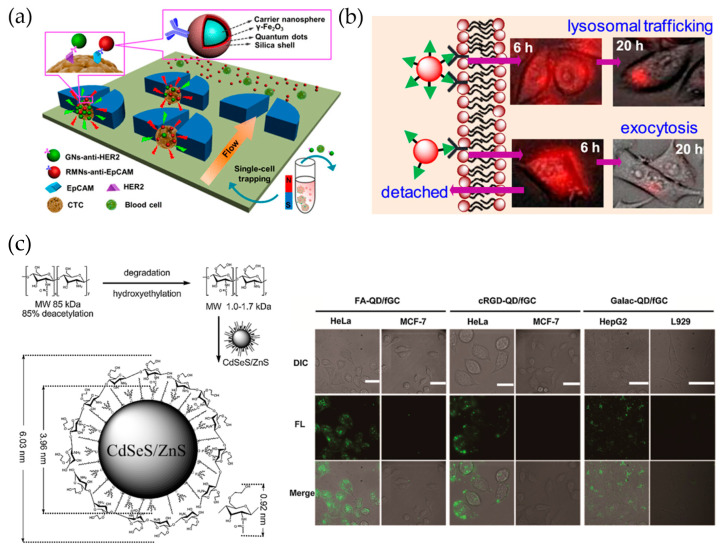
(**a**) Fabrication of IgG-QD@SiO_2_ conjugate via surface modification of QD@SiO_2_ and indirect immunoassay with IgG-QD@SiO_2_ conjugate [68]. Reprinted with permission from Anal. Chem. 2018, 90, 17, 10518–10526. Copyright 2018 American Chemical Society. (**b**) Cell uptake of QDs with different bioconjugation multivalencies [73]. Reprinted with permission from Langmuir 2019, 35, 35, 11380–11388. Copyright 2019 American Chemical Society. (**c**) Synthesis and use (live-cell imaging) of glycol chitosan-shelled QDs (QD/fGC) [74].

Even though profiling the heterogeneous phenotypes of individual circulating tumor cells (CTCs) from patients is challenging [67], research endeavors in this area will undoubtedly establish novel strategies for cancer management, especially in relation to personalized anticancer therapy. Wu et al. [68] reported an efficient and reliable chip-assisted multifunctional nanosphere system for analyzing the biomarker phenotypes of individual heterogeneous CTCs (Figure 5). The authors fabricated red- and green-emitting QDs that contained magnetic multifunctional nanospheres and CTC biomarker-targeting nanospheres, respectively. The fabricated nanospheres represented the optical properties of the corresponding QDs and were used for the simultaneous dual-fluorescence labeling and magnetic-tagging of CTCs. By integrating magnetic enrichment into a size-selective single cell-trapping microfluidic chip (SCT-chip), >90% of CTCs could be individually trapped and spatially separated from blood cells, even at concentrations as low as 10 CTCs per mL.

Bardi et al. [69] engineered and characterized NH_2_ functionalized CdSe/ZnS QD-doped silica NPs with both imaging and gene carrier capabilities. The QD-doped silica NPs were internalized by primary cortical neural cells without inducing cell death both in vitro and in vivo. Moreover, the abilities to bind, transport, and release DNA into the cell facilitated the GFP-plasmid transfection of NIH-3T3 and human neuroblastoma SH-SY5Y cells.

Because cancer metastasis mainly occurs through the lymph nodes, the ability to pinpoint the location of a metastatic lymph nodes is indispensable [70,71]. To locate metastatic lymph nodes, QDs can be injected intradermally (on the footpad of a mouse) and then imaged after they accumulate in the lymph nodes.

Erogbogbo et al. [72] reported that biocompatible silicon QDs, which possess desirable physical and optical properties and surface chemistry, covalently attached to a variety of biomolecules (e.g., lysine, folate, antimesothelin, and transferrin) and were selectively taken up by cancer cells. These findings contribute to the preclinical evaluation of silicon QDs and further demonstrate their potential as imaging agents in cancer applications and as a viable candidate for use in long-term and real-time cellular labeling and bioimaging.

Dalal and Jana [73] synthesized riboflavin (RF)-functionalized QDs by controlling RF multivalencies of RF: QD(RF)15, QD(RF)30, and QD(RF)70 (Figure 5b). The uptake of the resulting QDs by RF receptor-overexpressing KB cells revealed that the interaction between QD(RF) and target cells increased with increasing multivalency. In addition, this increase shifted the cell uptake mechanism from caveolae-clathrin to exclusively clathrin-mediated endocytosis and enhanced lysosomal trafficking. Based on these findings, the authors suggested that the multivalencies of conjugated biomolecules on the surface of nanoparticles should be adjusted to optimize imaging.

Zhao et al. [74] reported a method for the synthesis of glycol chitosan (GC)-shelled QD (QD/fGC) for live-cell imaging. After degradation and hydroxyethylation, modified GC was attached to the surface of QDs (Figure 5c), and the resulting QD/fGC particles exhibited excellent properties, which included good water solubility, high colloidal stability, and low non-specific adsorption. Bioactive molecules, such as folic acid, cRGD peptide derivative, and galactose, could be conjugated with QD/fGC via EDC coupling. These modified QD/fGCs were successfully selectively labeled with live HeLa cells.

#### 3.3.2. In Vivo Cell Tracking

As mentioned above, the remarkable optical properties of QDs provide critical advantages for monitoring the locations of injected cells in vivo, and highly sensitive QD-based in vivo tracing provides a simple approach and rapid method that does not require genetic modification.

**Figure 6:**
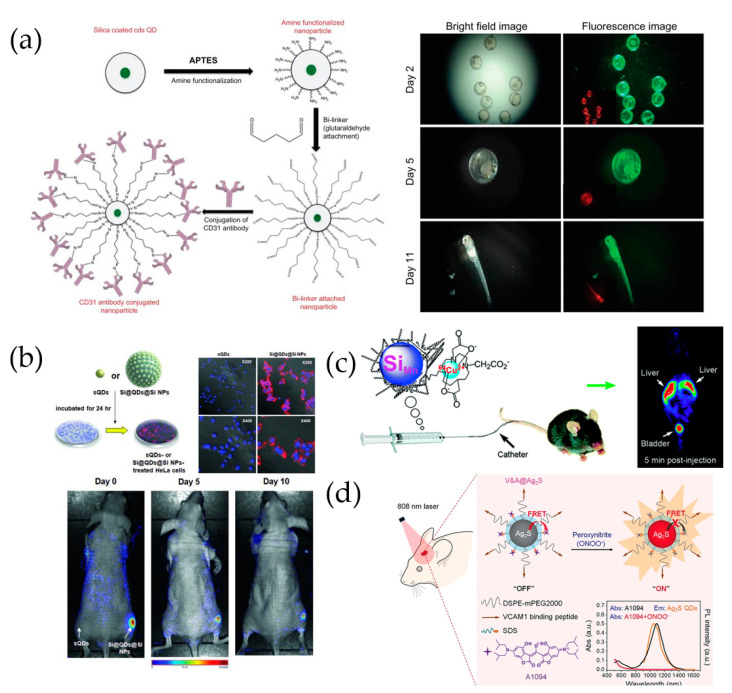
(**a**) Antibody conjugation onto silica-coated CdS QDs and in vivo fluorescence imaging of silica-coated CdS QD-treated embryos [75]. (**b**) In vivo cellular uptake of sQDs and SiO_2_@QDs@ SiO_2_ NPs [37]. (**c**) Use of dextran-coated silicon QDs in PET imaging and biodistribution [77]. Reprinted with permission from ACS Med. Chem. Lett. 2011, 2, 4, 285–288. Copyright 2011 American Chemical Society. (**d**) V&A@Ag_2_S probe preparation and in vivo detection of peroxynitrite [78].

Veeranarayanan et al. [75] described the synthesis of aqueous CdS-QDs and silica-encapsulated CdS-QDs using reverse microemulsion (Figure 6a), as well as their use as targeted optical probes with superior biocompatibility and photostability for in vivo live-cell imaging. Bare QDs were coated with silica to effectively suppress their cytotoxicity. This approach demonstrated exceptional in vivo biocompatibility and targeting precision against medaka fish embryos. The survival rate of the embryos treated with the silica-coated QDs was >90%, whereas that of embryos treated with bare CdS-QDs was close to 100%.

Intensely bright QD-based probes that consisted of QDs on 120 nm silica NPs with silica shells were investigated for their applicability to effective bioimaging, in terms of brightness and biocompatibility [37]. Silica-coated QD-embedded silica NPs (SiO_2_@QDs@ SiO_2_ NPs) that contained QDs with CdSe@ZnS (core-shell) were prepared. The PL of the SiO_2_@QDs@ SiO_2_ NPs was ~200 times greater than that of single QDs, and the NPs were also less toxic than equivalent numbers of silica-free single QDs. The outstanding in vivo applications of the SiO_2_@QDs@ SiO_2_ NPs were further demonstrated by the significantly enhanced fluorescence signals obtained from SiO_2_@QDs@ SiO_2_ NP-tagged cells implanted in mice (Figure 6b), when compared to those of SiO_2_@QDs@ SiO_2_ NPs, thereby indicating that SiO_2_@QDs@SiO_2_ NPs are useful in biomedicine and especially for in vivo cell tracking where high sensitivity is required.

May et al. [76] discovered that Pluronic (a nonionic surfactant) block copolymers could be used to encapsulate QDs@SiO_2_ NPs and make them water-dispersible and, thus, suitable for cancer imaging. Micelle-encapsulated QDs@SiO_2_ NPs that were injected intravenously into BALB/c mice, were preferentially taken up by the spleen, with some of them seen in the liver, but not by the heart, lungs, kidneys, nor brain.

Multimodal imaging materials and techniques have been proposed for the simultaneous collection of positron emission tomography (PET) and fluorescence images using a single nanomaterial. Whole-body (macroscale) imaging was performed using PET, and fluorescence was used to determine the distribution of isotopes at a microscopic level. Therefore, it is possible to exploit the unique properties of PET as well as the complementary information provided by fluorescence. A macrocyclic ligand-^64^Cu^2+^ complex was synthesized by Tu et al. [77] and used to label dextran-coated silicon QDs (mean hydrodynamic diameter of 15.1 ± 7.6 nm; Figure 6c). The chelate exhibited exceptional stability, as demonstrated by the absence of radiolabels under a ligand competition reaction with ethylenediaminetetraacetic acid (EDTA). The biodistribution of QDs in mice was quantitatively evaluated using in vivo PET and ex vivo gamma counting, which revealed that, even though the radiolabels were rapidly excreted via renal filtration, they also accumulated in the liver. The rapid clearance of dextran-coated Si QDs from the mouse bloodstream can inspire and provide useful information for the future design of QDs and NPs for biomedical applications.

Aptamers and cancer-tracing siRNA drugs can be loaded onto QDs and used to treat cancer cells in vivo. In this study, QDs were used as a drug delivery vehicle that could also be monitored using fluorescence imaging. Bagalkot et al. [79] developed a clever technology for linking siRNA-aptamer chimeras to carrier NPs and, in the process, resolved several technical hurdles previously encountered in chimera delivery, such as endosome escape and aptamer orientation control. The NPs possessed large surface areas, which facilitated high siRNA payloads and simultaneously exposed the aptamer for specific targeting, provided a proton sponge effect for endosome escape, and emitted fluorescence for imaging and quantification. The conventional siRNA-aptamer chimeras on NPs produced using one-step adsorption with random orientations and conformations did not elicit the much-improved RNAi effect observed for non-targeted NP-siRNA complexes (6–8% improvement in total cell population). However, the approach promoted selective gene silencing and yielded 34% more silenced cells in the total cell population over non-targeted NP-siRNA complexes, for the same RNA concentration.

Polymeric micelles for efficient theranostics were fabricated and exploited to encapsulate both an antitumor drug (doxorubicin) and Au core-shell QD NPs (Au-SiO_2_/QDs) [80]. α,β-Poly(N-hydroxyethyl)-dl-aspartamide (PHEA) was functionalized using lipoic acid (LA), PEG, and folic acid (FA) pendant moieties to derive PHEA-LA-PEG-FA micelles, which were capable of self-assembly in an aqueous medium, forming polymeric micelles, and carrying both targeting groups (FA) and hydrophilic chains (PEG) on their surfaces. The drug-carrying ability and cytotoxicity of the PHEA-LA-PEG-FA micelles against breast cancer cells were characterized using doxorubicin as a model anticancer drug. The cell-imaging and photo-thermal anticancer treatment properties of the micelle-incorporated Au-shell QDs were also assessed.

Li et al. [78] reported the fabrication and application of an Ag_2_S quantum dot-based near-infrared window (NIR-II) nanoprobe for the in vivo early real-time diagnosis of traumatic brain injury (TBI; Figure 6d). The authors fabricated a targeted activatable fluorescent nanoprobe (V&A@Ag_2_S), which is a vascular cell adhesion molecule 1 (VCAM1)-binding peptide for endocytosis and the A1094 chromophore for FRET. The V&A@Ag_2_S nanoprobe was turned off as a result of the FRET effect with A1094. After intravenous injection, V&A@Ag_2_S accumulated in inflamed vascular endothelia associated with TBI via VCAM1-mediated endocytosis, and A1094 was bleached as a result of the presence of peroxynitrite, which is a prodromal TBI biomarker. With bleaching of A1094, the NIR-II fluorescence of V&A@Ag_2_S was recovered, and real-time in vivo bioimaging was enabled.

Even though QDs often incorporate components that are critically detrimental to cells in vivo, they should still be studied thoroughly to elucidate their remarkable advantages, and such research efforts could potentially lead to the development of nontoxic QDs.

## 4. Conclusions and Perspective

Quantum dots have a wide variety of current and potential applications. When compared to conventional organic dyes, QDs exhibit a range of unique physiochemical properties and excellent optical properties. In particular, distinct QDs can be excited by a single light source and yet emit separate colors across a broad spectral range and with minimal spectral overlap. Such properties make QDs promising nanomaterials for biomedical applications, especially for multiplex imaging. The physiochemical and optical properties of QDs, such as a broad absorption band, long first exaction radiative lifetime, small Stokes shift, and ability to tune semi-conductor core particle aspect ratios to obtain linearly polarized PL emissions also make them potentially useful to a variety of scientific and industrial settings, including agriculture, environmental science, biology, biosensing, in vitro assays, and imaging. However, efficient QDs are typically prepared as hydrophobic particles that are unsuitable for bioapplications, and cytotoxicity can hinder the use of Cd-based QDs.

As a potential solution, silica coating has been used to circumvent many of the key problems in regard to QD bioapplications, and silica-coated QDs can be used in the near future for a wide range of biological and medical applications and investigations. In particular, silica-coated QDs can be expected to be utilized and commercialized by the medical fields associated with the world’s rapidly growing aging population.

Importantly, the application of silica-coated QDs still faces a variety of limitations, including issues with biosafety and surface modification for biological applications. The inevitable shortcomings of silica-coated QDs for in vivo studies stem from the application of Cd, and the in vivo toxicity and fate of branched QDs (e.g., nontoxic or graphene QDs) remain contentious. Furthermore, nontoxic QDs also continue to fall short of the quantum efficiencies of Cd-based QDs. Nevertheless, further research of both QD synthesis and practical bioapplications could resolve these existing problems, and both direct and indirect breakthroughs in the biological and medical fields are anticipated to result from studies of silica-based QDs.

## Data Availability

Not applicable.
